# Dual anti-CD20/anti-CD38 therapy for severe recurrent FSGS with longitudinal anti-nephrin assessment

**DOI:** 10.1093/ndt/gfag088

**Published:** 2026-04-15

**Authors:** Xhuliana Kajana, Agnese Spennacchio, Francesca Chiara Viazzi, Carolina Bigatti, Pasquale Esposito, Angelica Parodi, Gianluca Caridi, Daniela Picciotto, Maria Teresa Gandolfo, Gabriele Mortari, Giovanni Varotti, Katia Mazzocco, Enrico Verrina, Alberto Magnasco, Paolo Cravedi, Andrea Angeletti

**Affiliations:** Department of Pediatric Medical Sciences, Nephrology, Dialysis and Transplantation Unit, IRCCS Istituto Giannina Gaslini, Genoa, Italy; Department of Pediatric Medical Sciences, Nephrology, Dialysis and Transplantation Unit, IRCCS Istituto Giannina Gaslini, Genoa, Italy; Department of Internal Medicine and Medical Specialties, Unit of Nephrology, Dialysis and Transplantation, IRCCS Azienda Ospedaliera Metropolitana (IRCCS AOM) San Martino, Genoa, Italy; Department of Internal Medicine and Medical Specialties (DIMI), University of Genova, Genova, Italy; Department of Pediatric Medical Sciences, Nephrology, Dialysis and Transplantation Unit, IRCCS Istituto Giannina Gaslini, Genoa, Italy; Department of Internal Medicine and Medical Specialties, Unit of Nephrology, Dialysis and Transplantation, IRCCS Azienda Ospedaliera Metropolitana (IRCCS AOM) San Martino, Genoa, Italy; Department of Internal Medicine and Medical Specialties (DIMI), University of Genova, Genova, Italy; Department of Internal Medicine and Medical Specialties, Unit of Nephrology, Dialysis and Transplantation, IRCCS Azienda Ospedaliera Metropolitana (IRCCS AOM) San Martino, Genoa, Italy; Department of Pediatric Medical Sciences, Nephrology, Dialysis and Transplantation Unit, IRCCS Istituto Giannina Gaslini, Genoa, Italy; Department of Internal Medicine and Medical Specialties, Unit of Nephrology, Dialysis and Transplantation, IRCCS Azienda Ospedaliera Metropolitana (IRCCS AOM) San Martino, Genoa, Italy; Department of Internal Medicine and Medical Specialties, Unit of Nephrology, Dialysis and Transplantation, IRCCS Azienda Ospedaliera Metropolitana (IRCCS AOM) San Martino, Genoa, Italy; Department of Pediatric Medical Sciences, Nephrology, Dialysis and Transplantation Unit, IRCCS Istituto Giannina Gaslini, Genoa, Italy; Transplant Surgery Unit, IRCCS San Martino Polyclinic Hospital, Genoa, Italy; Pathology Unit, IRCCS Giannina Gaslini Institute, Genoa, Italy; Department of Pediatric Medical Sciences, Nephrology, Dialysis and Transplantation Unit, IRCCS Istituto Giannina Gaslini, Genoa, Italy; Department of Pediatric Medical Sciences, Nephrology, Dialysis and Transplantation Unit, IRCCS Istituto Giannina Gaslini, Genoa, Italy; Translational Transplant Research Center, Department of Medicine, Icahn School of Medicine at Mount Sinai, New York, NY, USA; Department of Pediatric Medical Sciences, Nephrology, Dialysis and Transplantation Unit, IRCCS Istituto Giannina Gaslini, Genoa, Italy

To the Editor,

Primary focal segmental glomerulosclerosis (FSGS) recurs in approximately 30%–40% of patients after kidney transplantation and is associated with early graft dysfunction and accelerated graft loss. Its pathophysiology remains incompletely understood, and no approved therapies are available. Accumulating evidence, such as the clinical efficacy of therapies targeting B cells and long-lived plasma cells, implicates antibodies and/or B cell/plasma cell secreting factors in disease pathogenesis [1].

Autoantibodies directed against the glomerular slit diaphragm, including nephrin, have been proposed as potential mediators of post-transplant recurrence [2–7]. In addition, natural immunoglobulin M (IgM) targeting endothelial antigens may also play a role in FSGS progression [8], but data in post-transplant FSGS recurrence (rFSGS) remain limited.

Here, we report a case of early rFSGS associated with anti-nephrin antibody that was successfully treated by combined B-cell and plasma-cell targeting therapy.

A 27-year-old patient with ESKD secondary to FSGS, negative for known monogenic variants of FSGS and presenting as frequently relapsing and multidrug-dependent FSGS, received a kidney transplant from a deceased donor. Induction immunosuppression consisted of anti-thymocyte globulin (3.5 mg/kg) and steroids, followed by maintenance therapy with steroids, tacrolimus and mycophenolate mofetil. Urine output recovered immediately after transplant, but early-onset of severe, nephrotic-range proteinuria (28.5 g/day) developed on post-transplant Day 1. On Day 3, the patient developed oliguria followed by anuria, requiring hemodialysis (HD) on Day 5. The following day, a severe peri-graft bleeding occurred during HD: imaging with ultrasound and computed tomography revealed a large and persistent perirenal hematoma (20 × 5 cm). Due to hemodynamic instability and poor graft accessibility related to the extensive perirenal collection, a kidney biopsy was contraindicated. Doppler ultrasound demonstrated preserved graft vascularization with diffusely increased intrarenal resistive indices (0.7–0.8). Recurrent FSGS, acute ischemic injury secondary to severe hypoperfusion and acute rejection were all considered plausible contributors to the persistent anuria.

However, serum samples collected the day before transplant and on post-operative Day 1 were retrospectively analyzed after the onset of anuria and tested positive for anti-nephrin antibodies using enzyme-linked immunosorbent (ELISA) and immunoprecipitation assays, as previously described [9] (see online Supplementary Material). In the absence of risk factors for acute rejection (donor-specific antibodies were negative), we interpreted the clinical course secondary to a severe rFSGS, despite the lack of a histological documentation because contraindicated. Therefore, plasma exchange (PEX) and anti-CD20 therapy with the humanized monoclonal antibody obinutuzumab was initiated (Fig. 1; see online Supplementary Material for clinical details). To drain the perirenal hematoma, the patient underwent two surgical re-interventions and multiple blood transfusions.

**Figure 1:**
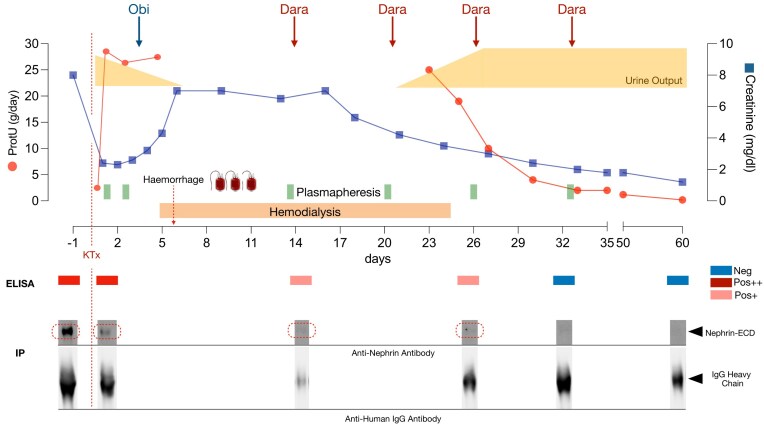
Longitudinal clinical, biochemical and serological course following kidney transplantation. Temporal relationship between proteinuria (ProtU, g/day), serum creatinine (mg/dL), urine output, renal replacement therapy, therapeutic interventions and anti-nephrin antibody levels during the early post-transplant period. Immediate post-transplant nephrotic-range proteinuria and subsequent anuria were followed by hemorrhagic complications requiring surgical re-interventions and HD. PEX sessions and administration of obinutuzumab (Obi, anti-CD20) and daratumumab (Dara, anti-CD38) are indicated. Longitudinal serological assessment by ELISA and immunoprecipitation (IP) shows the progressive decline and eventual negativization of anti-nephrin antibodies, paralleling reduction in proteinuria and recovery of graft function. KTx, kidney transplantation.

Over this period, PEX was continued (for a total of six treatments) and anti-CD38 monoclonal antibody daratumumab was initiated based on our prior data showing the efficacy of anti-CD20/anti-CD38 therapy in rFSGS [10–12]. On post-operative Day 21, urine output recovered, followed by improvement in kidney function from Day 24, which allowed discontinuation of dialysis. Serial measurements of anti-nephrin antibody (always measured prior to PEX and monoclonal infusions) showed a progressive decline. PEX and daratumumab infusions (administered at a distance from PEX), were continued until Day 33, when serum anti-nephrin antibody became definitively negative (Fig. 1). At Day 60 post-transplantation, proteinuria is 0.2 g/day, eGFR is 76 mL/min/1.73 m^2^ and anti-nephrin antibodies are undetectable in the circulation.

This is the first reported case of rFSGS in whom combined PEX, anti-CD20 and anti-CD38 therapy was guided by anti-nephrin antibody. Despite the dramatic course of the disease, such treatment safely promoted a full recovery of graft function.

Anti-nephrin antibody positivity supported the diagnosis of rFSGS even in the absence of a histological documentation of rFSGS and guided the duration of treatment. In this patient, serial monitoring of such antibodies is currently ongoing to detect relapses early and allow for timely treatment.

The combined use of anti-CD20 and anti-CD38 therapies does not allow us to conclude whether the source of anti-nephrin antibodies was B cells or plasma cells. Also, the fact that changes in anti-nephrin and total IgG followed similar trends leaves open the possibility that IgG with other targets played a pathogenic role in this patient. Intriguingly, IgM were also rapidly depleted, suggesting that also this immune globulin might have played a role in disease activity (Fig. 2A), as previously reported in native disease [13]. In our biobank, we had stored serum samples collected throughout the patient’s clinical course (the diagnosis was made at 6 years of age). To further examine the association between anti-nephrin antibodies and disease activity, we quantified these antibodies in seven samples obtained between the ages of 9 and 19 years, during periods of active disease or remission and at different time points related to the immunosuppressive therapy (Fig. 2B). Immunofluorescence staining on native kidney biopsy, performed at the age of 9 years, showed no IgG deposition (Supplementary data, Fig. S1).

**Figure 2:**
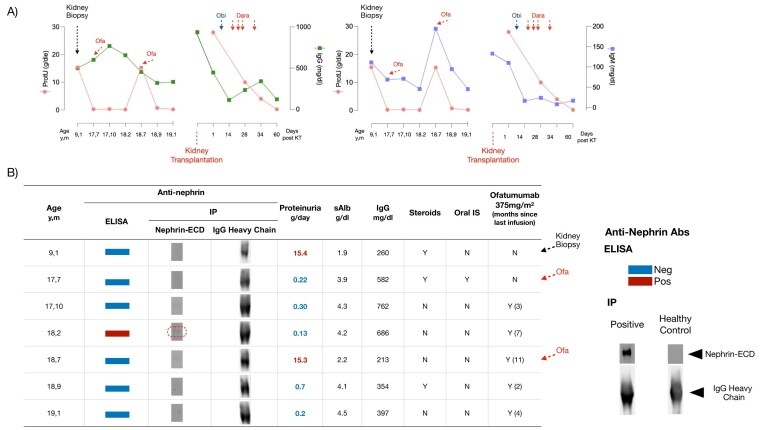
IgG, IgM trends and anti-nephrin antibody trends during native kidney disease and after kidney transplantation. (**A**) Serial changes in proteinuria and serum immunoglobulin levels, highlighting a closer temporal association with IgM than with total IgG, in the pre-transplant phase. (**B**) Longitudinal analysis of anti-nephrin antibody levels during native kidney disease, evaluated by ELISA and immunoprecipitation, showing dissociation from clinical disease activity.

Notably, only one sample tested positive for anti-nephrin IgG, without a clear correlation between antibody presence and disease activity. This finding suggests that fluctuations in serum anti-nephrin antibody levels may occur independently of clinical activity, particularly in the later phases of the disease. Interestingly, the pre-transplant serum sample was strongly positive, despite the patient having been on HD for more than 2 years.

An additional hypothesis proposed by others is that anti-nephrin antibodies may arise secondary to other slit diaphragm–targeting antibodies through molecular epitope spreading [5, 14, 15]. Overall, these data suggest that repeated measurements may be warranted in the immediate pre-transplant period in cases where suspicion for an autoimmune pathophysiology remains high. However, these observations should be interpreted with caution, as ELISA-based assays for anti-nephrin antibodies have been reported to show variable sensitivity and specificity [9], which may partly contribute to the apparent inconsistency.

The doses of obinuzuzumab and daratumumab we used are significantly lower than those adopted for hematological indications. The effective levels of these biologics might be further reduced by urinary losses in the presence of proteinuria. As a result, the excellent treatment efficacy was associated with a very good tolerability. Future studies are needed to establish whether even lower doses are enough to obtain disease remission.

Together, this case documents that combined B-cell and plasma-cell depletion can promote remission even in severe rFSGS cases. When present, anti-nephrin antibodies may provide an important biomarker of disease activity, especially when renal biopsy is not feasible or in the presence of anuria. Notably, in this case, anti-nephrin antibody assessment was performed retrospectively after transplantation and therefore did not influence the initial transplant decision. These findings highlight the need for prospective evaluation of anti-nephrin antibodies in pre-transplant risk stratification and raise the testable hypothesis that their identification before transplantation could inform tailored induction strategies in selected high-risk patients.

## Supplementary Material

gfag088_Supplemental_File

## References

[1] Uffing A, Pérez-Sáez MJ, Mazzali M et al. Recurrence of FSGS after kidney transplantation in adults. Clin J Am Soc Nephrol. 2020;15:247–56. 10.2215/CJN.08970719 31974287 PMC7015092

[2] Watts AJB, Keller KH, Lerner G et al. Discovery of autoantibodies targeting nephrin in minimal change disease supports a novel autoimmune etiology. J Am Soc Nephrol. 2022;33:238–52. 10.1681/ASN.2021060794 34732507 PMC8763186

[3] Shirai Y, Miura K, Ishizuka K et al. A multi-institutional study found a possible role of anti-nephrin antibodies in post-transplant focal segmental glomerulosclerosis recurrence. Kidney Int. 2024;105:608–17. 10.1016/j.kint.2023.11.022 38110152

[4] Hattori M, Shirai Y, Kanda S et al. Circulating nephrin autoantibodies and posttransplant recurrence of primary focal segmental glomerulosclerosis. Am J Transplant. 2022;22:2478–80. 10.1111/ajt.17077 35472030 PMC9790549

[5] Shirai Y, Miura K, Horita S et al. A case of post-transplant recurrent focal segmental glomerulosclerosis: graft biopsy showing IgG colocalization with Nephrin, Podocin, and Kirrel1. Kidney Int. 2025;108:714–5. 10.1016/j.kint.2025.06.012 40975534

[6] Batal I, Watts AJB, Gibier J-B et al. Pre-transplant anti-nephrin antibodies are specific predictors of recurrent diffuse podocytopathy in the kidney allograft. Kidney Int. 2024;106:749–52. 10.1016/j.kint.2024.07.022 39127225 PMC11416305

[7] Hengel FE, Dehde S, Lassé M et al. Autoantibodies targeting nephrin in podocytopathies. N Engl J Med. 2024;391:422–33. 10.1056/NEJMoa2314471 38804512

[8] Trachtman H, Laskowski J, Lee C et al. Natural antibody and complement activation characterize patients with idiopathic nephrotic syndrome. Am J Physiol Renal Physiol. 2021;321:F505–16. 10.1152/ajprenal.00041.2021 34459222 PMC8560405

[9] Liu P, Liu S, Dalal V et al. Evaluation of methodologies in anti-nephrin autoantibody detection. Kidney Int. 2025;108:485–90. 10.1016/j.kint.2025.05.018 40490077 PMC12354293

[10] Angeletti A, Bin S, Magnasco A et al. Efficacy of combined rituximab and daratumumab treatment in posttransplant recurrent focal segmental glomerulosclerosis. Am J Transplant. 2024;24:688–92. 38101474 10.1016/j.ajt.2023.12.010

[11] Cravedi P, Maggiore U, Mortari G et al. Combined anti-CD20/anti-CD38 therapy in posttransplant focal segmental glomerulosclerosis recurrence: a retrospective, international, multicenter study. Transplant Direct. 2026;12:e1908. 10.1097/TXD.0000000000001908 41567757 PMC12818861

[12] Randone P, Sanna E, Dolla C et al. Rescue with obinutuzumab and daratumumab as combined B cell/plasma cell targeting approach in severe posttransplant focal segmental glomerulosclerosis recurrence. Am J Transplant. 2024;24:1896–900. 10.1016/j.ajt.2024.06.010 39029875

[13] Angeletti A, Bin S, Kajana X et al. Combined rituximab and daratumumab treatment in difficult-to-treat nephrotic syndrome cases. Kidney Int Rep. 2024;9:1892–6. 10.1016/j.ekir.2024.04.006 38899172 PMC11184257

[14] Raglianti V, Angelotti ML, De Chiara L et al. Anti-nephrin, anti-podocin and anti-Kirrel1 antibodies: biological challenges and clinical implications. Nephrol Dial Transplant. 2025;41:428–36. 10.1093/ndt/gfaf156 40815258

[15] Raglianti V, Angelotti ML, De Chiara L et al. Anti-slit antibodies against Podocin and Kirrel1 in pediatric and adult podocytopathies. J Am Soc Nephrol. 2025;36:702–5. 10.1681/ASN.0000000642 39883528 PMC11975239

