# Supplementary material for: Dual anti-CD20/anti-CD38 therapy for severe recurrent FSGS with longitudinal anti-nephrin assessment
Source: Nephrol Dial Transplant. 2026 Apr 15;41(8):1577–80. doi: 10.1093/ndt/gfag088 (PMC13423821; doi:10.1093/ndt/gfag088)
Supplement: gfag088_Supplemental_File [file gfag088_supplemental_file.docx]

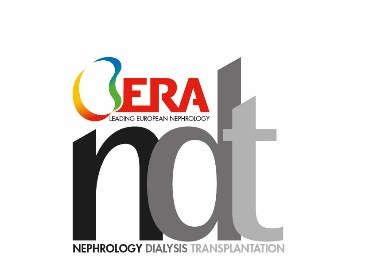


**Supplementary Material**

**Title: Dual Anti-CD20/Anti-CD38 Therapy for Severe Recurrent FSGS with Longitudinal Anti-Nephrin Assessment**

**Authors:** Xhuliana Kajana^1^, Agnese Spennacchio^1^, Francesca Chiara Viazzi^2,3^, Carolina Bigatti^1^, Pasquale Esposito^2,3^, Angelica Parodi^2^, Gianluca Caridi^1^, Daniela Picciotto^2^, Maria Teresa Gandolfo^2^, Gabriele Mortari^1^, Giovanni Varotti^4^, Katia Mazzocco^5^, Enrico Verrina^1^, Alberto Magnasco^1^, Paolo Cravedi^6^* and Andrea Angeletti^1^*

**Affiliations:** *^1^Nephrology, Dialysis and Transplantation Unit, IRCCS Istituto Giannina Gaslini, Genoa, Italy; ^2^Unit of Nephrology, Dialysis and Transplantation, IRCCS Azienda Ospedaliera Metropolitana (IRCCS AOM) San Martino, Genoa, Italy; ^3^Department of Internal Medicine and Medical Specialties (DIMI), University of Genova, Genova, Italy; ^4^Transplant Surgery Unit, IRCCS San Martino Polyclinic Hospital, Genoa, Italy; ^5^Pathology Unit, IRCCS Giannina Gaslini Institute, Genoa, Italy; ^6^Translational Transplant Research Center, Department of Medicine, Icahn School of Medicine at Mount Sinai, New York, NY*

*These Authors contributed equally as senior authors

Table of contents

1. Supplementary Methods …………………………………..………………………..…… 2
2. Supplementary Results……………………………………………………………………5
3. Supplementary References……………………………………………………………….7

This supplementary material has been provided by the authors to give readers additional

information about their work.

**1. Supplementary Methods**

*1.1. Participant*

Written informed consent was obtained from the patient and transplantation was performed in accordance with the Declaration of Istanbul.

*1.2 Treatments*

Obinutuzumab (1000 mg) and Daratumumab (1,200mg) were administered intravenously. Patient received a premedication before each infusion with methyl-prednisolone (2 mg/kg infused in 30’ intravenous diluted in 100 ml of normal saline), oral cetirizine (0.2 mg/kg), and oral paracetamol (15 mg/kg). Obinutuzumab was diluted in 500 ml of normal saline and administered at 9 ml/h for the first 30’; thereafter, the infusion rate was doubled every 30 minutes up to a maximum of 72 ml/h. Daratumumab was diluted in 1000 ml of normal saline and administered at 9 ml/h for the first 30’; thereafter, the infusion rate was doubled every 30 minutes up to a maximum of 72 ml/h.

1.4 *Anti-Nephrin Antibodies*

The presence of anti-nephrin autoantibodies was determined by immunoprecipitation, as previously reported,^S1^ using recombinant human Nephrin protein (NPHS1-ECD aa23-1055 produced using human-derived HEK293 cells, Cat# 17757-H08H, Sino Biological). 3μL of patient serum and serum from healthy individuals was incubated with 100 ng NPHS1-ECD antigen in 100 μL TBST overnight, at constant rotation at 4 °C. After overnight incubation, 15 μL of protein G magnetic beads (Cytiva, Cat# 28951379) was added and incubated for 4 hours at constant rotation at room temperature. The magnetic beads were then washed 5 times with TBST and the immune-complex was eluted with 15 μL Laemmli buffer under denaturing conditions and incubated at 95 °C for 3 min. Samples were electrophoresed in a gradient gel (4-15%) by SDS-PAGE and transferred to nitrocellulose membrane (BioRad, Cat# 1620112) under semi-dry conditions. Membrane was blocked with 5% milk for 1 hour at room temperature and then incubated overnight at 4 °C with sheep anti-NPHS1 polyclonal antibody (1: 800 diluted, R&D Systems, Cat#HAF016) in blocking buffer. The membrane was washed 3 times with TBST and then incubated with HRP-conjugated donkey anti-sheep IgG secondary antibody (R&D, Cat# HAF016) for 2 hours at room temperature. After three washes with TBST, membrane was incubated with SuperSignal™ West Pico PLUS Chemiluminescent Substrate (Thermo Scientific™, Cat #34580) and imaged by ChemiDoc™ Imaging System. The membrane was then washed and incubated with horseradish peroxidase–conjugated anti-human IgG secondary antibody (Sigma, Cat# AP101P) for 2 hours. After three washes with TBST, membrane was incubated with chemiluminescent substrate and imaged.

For ELISA, high-binding 96-well plates were coated overnight at 4 °C with recombinant human nephrin extracellular domain (NPHS1-ECD–His; 100 ng/well; amino acids 23–1055) diluted in phosphate-buffered saline (PBS). Uncoated wells were included to assess non-specific binding. Plates were washed with Tris-buffered saline containing 0.1% Tween-20 (TBST) and blocked for 1 hour at room temperature with 5% non-fat dry milk in TBST. Serum samples were diluted 1:100 in blocking buffer and incubated for 2 hours at room temperature. After washing, plates were incubated with horseradish peroxidase–conjugated anti-human IgG secondary antibody for 1 hour at room temperature. Signal was developed using tetramethylbenzidine substrate and stopped with sulfuric acid, and optical density was measured using a microplate reader.

As previously described^S2^, a standard curve for anti-nephrin IgG detection was generated using a highly nephrin-positive serum, arbitrarily assigned a value of 1000 relative units (RU)/mL. Anti-nephrin antibody titers in serum samples were derived from this standard curve. Results are reported in Supplementary Table 1.

*1.5 Immunofluorescence staining*

Immunofluorescence staining was performed on native kidney biopsy sections using standard protocols. Briefly, Tissue section (5 µm) was deparaffinized using Histo-C histological clearing agent and rehydrated prior to antigen retrieval in 0.01 mol/L citric acid buffer (pH 6.4) at 96 °C for 24 minutes. Sample was then incubated in a blocking solution containing 5% BSA for 30 minutes at room temperature. Section was incubated overnight at 4 °C with a polyclonal anti-Nefrin primary antibody (NBP1-77303, NovusBio) diluted 1:200 in 1% BSA, followed by three washes in PBS 1× containing 0.1% Triton X-100.

Subsequently, slide was incubated for 1 hour at room temperature in the dark with a goat anti-rabbit Alexa Fluor 568 secondary antibody (ThermoFisher) diluted 1:100 in 1% BSA, and an anti-human IgG antibody (F0202, Polyclonal Rabbit Anti-Human/FITC, Dako) diluted 1:100 in 1% BSA. After three additional washes in PBS 1× with 0.1% Triton X-100, section was mounted with Vectashield (Vector Laboratories). Images were acquired using a Zeiss Axio Imager M2 microscope.

2**. Supplementary Results**

*Supplementary Table 1. Anti-nephrin antibody titers*

|  | IgG anti-nephrin (RU/ml) |
| --- | --- |
| Native Disease, age (y,m) | |
| 9,1 | 0.00 |
| 17,7 | 0.00 |
| 17,10 | 0.00 |
| 18,2 | 0.791 |
| 18,7 | 0.00 |
| 18,9 | 0.00 |
| 19,1 | 0.00 |
| Kidney Transplantation, day | |
| Day -1 | 365.6 |
| Day 1 | 233.3 |
| Day 14 | 112.2 |
| Day 26 | 0.562 |
| Day 32 | 0.00 |
| Day 60 | 0.00 |

*Supplementary Figure 1
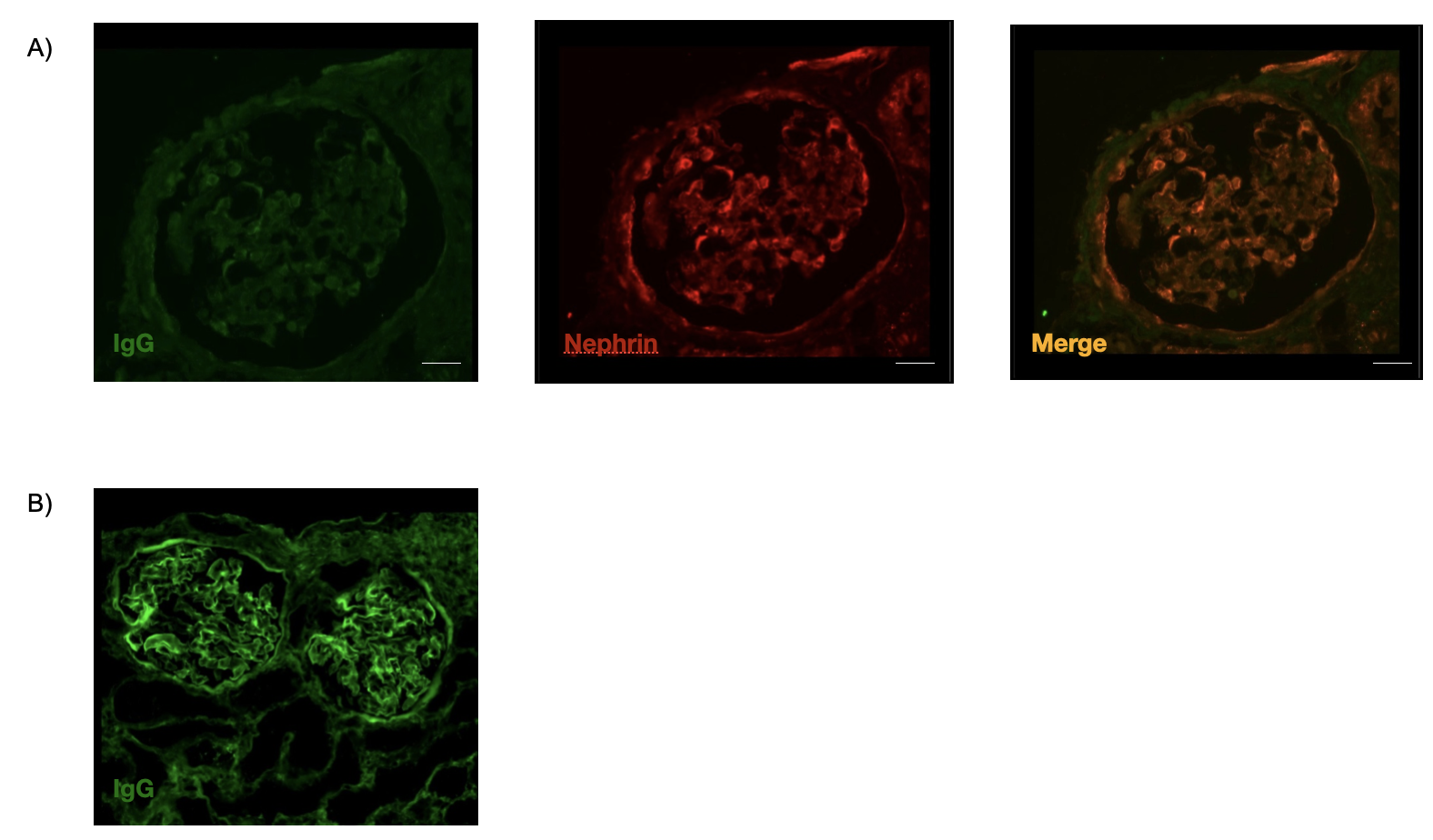
*

A) Nephrin staining (red) was preserved along the glomerular capillary loops, whereas no IgG deposition (green) was detected in the patient’s native kidney biopsy (40x). B) As a positive control, a lupus nephritis biopsy with known IgG deposition was included and processed in parallel (20x).

**3. Supplementary References**

S1. Evaluation of Methodologies in Anti-nephrin Autoantibody Detection. Liu P, Liu S, Dalal V, Lane J et al. Kidney Int. 2025 Sep;108(3):485-490. doi: 10.1016/j.kint.2025.05.018.

S2. Watts AJB, Keller KH, Lerner G*, et al.* Discovery of Autoantibodies Targeting Nephrin in Minimal Change Disease Supports a Novel Autoimmune Etiology. *J Am* Soc Nephrol 2022; **33:** 238-252.
